# 14-3-3σ gene silencing during melanoma progression and its role in cell cycle control and cellular senescence

**DOI:** 10.1186/1476-4598-8-53

**Published:** 2009-07-30

**Authors:** Julia Schultz, Saleh M Ibrahim, Julio Vera, Manfred Kunz

**Affiliations:** 1 Department of Cardiac Surgery, University of Rostock, 18055 Rostock, Germany; 2 Department of Dermatology, Allergology and Venereology, University of Lübeck, 23538 Lübeck, Germany; 3 Department of Computer Science, University of Rostock, 18051 Rostock, Germany; 4 Comprehensive Center for Inflammation Medicine, University of Lübeck, 23538 Lübeck, Germany

## Abstract

**Background:**

The family of 14-3-3 proteins plays an important role in cancer biology by interfering with intracellular signalling pathways and cell cycle checkpoints. The 14-3-3σ isoform acts as a tumor suppressor and is often inactivated during tumor development.

**Results:**

Here, we demonstrate enhanced CpG methylation of the 14-3-3σ gene in lymph node and cutaneous melanoma metastases compared with primary tumors, associated with dramatically reduced mRNA expression. In line with this, treatment of different metastatic melanoma cell lines with 5-aza-2'-deoxycytidine (5-Aza-CdR), a potent inhibitor of cytosine methylation, significantly induces 14-3-3σ protein expression. Additional treatment with histone deacetylase inhibitor 4-phenylbutyric acid (Pba) further enhances 14-3-3σ expression. Induction of 14-3-3σ expression by 5-Aza-CdR/Pba treatment leads to almost complete inhibition of cell proliferation, with cells predominantly arrested in G2-M. The antiproliferative effect of 5-Aza-CdR/Pba was reversed in 14-3-3σ knockdown cells. Similarly, melanoma cell lines stably overexpressing 14-3-3σ show dramatically reduced cell proliferation rates. Moreover, synchronous 14-3-3σ stably overexpressing cells do not progress through cell cycle, but display a permanent increase in the population of 4*n *DNA containing cells. Interestingly, overexpression of 14-3-3σ induces senescence of melanoma cells and is involved in melanoma cell senescence under genotoxic stress. Finally, 14-3-3σ knockdown supports migratory capacity of melanoma cells *in vitro*, while 14-3-3σ overexpression has opposing effects.

**Conclusion:**

Taken together, the present report indicates that epigenetic silencing of 14-3-3σ might contribute to tumor progression in malignant melanoma via loss of cell cycle control, impaired cellular senescence program and support of migratory capacity.

## Background

Malignant melanoma is a highly aggressive tumor with poor prognosis in the metastatic stage [1,2]. The molecular events underlying initial tumor development and further tumor progression are still poorly understood [2-4]. In a recent series of large-scale genetic studies, activating mutations were found in *BRAF *and *NRAS *oncogenes in a significant proportion of primary melanomas [5-8]. However, since also benign melanocytic nevi as classical melanoma precursor lesions showed a high percentage of activating *BRAF *mutations, further molecular mechanisms might contribute to primary tumor development [9,10]. In a more recent genome-wide RNA-interference screening targeting 28,000 genes, 17 genes were identified required to block uncontrolled proliferation of melanocytes in the presence of activated *BRAF *(BRAFV600E). Among these genes with tumor suppressor function, insulin-like growth factor binding protein 7 (*IGFBP7*) appears to play an outstanding role. Interestingly, the *IGFBP7 *gene is often silenced by epigenetic mechanisms in primary melanomas, and might thereby contribute to early malignant transformation of malignant melanoma. However, the functional relevance of these findings in human melanoma remains to be shown. Based on current findings, it appears that several mechanisms act together during malignant transformation and further progression of melanoma cells. Among these are activating or inactivating mutations of members of the Akt signalling pathway, namely phosphatidylinositol 3-kinase CA (*PI3KCA*), Akt kinase and the tumor suppressor phosphatase and tensin homolog (*PTEN*) [2,5].

Earlier reports showed that classical tumor suppressor molecules such as p16 and retinoblastoma protein might play role in malignant melanoma development [4]. It is well established that familial melanoma patients display mutations in the tumor suppressor protein p16 (*CDKN2A*) in almost half of the cases. In line with this, mice genetically engineered for p16 inactivation and overexpression of activated Ras develop primary melanomas and distant metastases [4]. However, only a small percentage of non-familial melanoma patients show inactivating mutations in the *CDKN2A *tumor suppressor gene. Further tumor suppressor molecules might thus be involved in melanoma suppression, the loss of which might give rise to primary tumors as well as metastases.

The family of 14-3-3 proteins comprises seven small molecules termed 14-3-3β, γ, ε, η, σ, τ, and ζ, which are expressed in a large variety of different tissues [11,12]. Earlier reports suggested that expression of 14-3-3σ was restricted to cells of epithelial origin, in particular stratified epithelia which led to the designation stratifin (*SFN*). 14-3-3 proteins act as phosphoserine- and phosphothreonine-binding proteins binding to the consensus motif RSXpS/TXP [13]. The list of interacting proteins comprises a large variety of different molecules, many of which are involved in intracellular signalling, apoptosis and cell cycle regulation, such as protein kinase C (PKC), RAF1, small G proteins, BAD, and CDC25C [11]. During interaction with RAF1, a member of the oncogene RAS/RAF/MEK/Erk pathway, dimeric 14-3-3 proteins exert a dual function. They maintain Raf1 in an inactive state in the absence of activated RAS, but stabilize the active conformation of Raf1 after pathway activation [14]. However, the exact mechanism of how RAF-1 is regulated by 14-3-3 proteins is still unknown. In general, 14-3-3 proteins are involved in cell cycle machinery at different checkpoints in order to allow DNA damage repair or to establish a permanent arrest of cells that have severe damage [12,15].

In more recent investigations using tandem affinity purification combined with multidimensional protein identification, 117 proteins were identified as putative interaction partners of 14-3-3σ [16]. Among these were 92 with known biological functions, many of which implicated in mitogenic signalling such as APC, A-RAF, B-RAF, and c-RAF, casein kinase II, PI3K-C2β, or cell cycle regulation such as AJUBA, WEE1, and c-TAK1. Together, these findings further emphasized the role of 14-3-3σ as a major regulator of cell cycle control [12,15].

There is an increasing body of evidence that inactivation of 14-3-3σ is involved in tumor development in a variety of malignant tumors [11]. First, 14-3-3σ is a well-known downstream target of tumor suppressor p53, which is inactivated in almost half of all cancers [17]. Second, it is involved in stabilization and activation of p53 in a positive feedback loop and overexpression of 14-3-3σ leads to reduced tumorigenicity of oncogene expressing NIH 3T3 cells [18,19]. Third, absence of 14-3-3σ is associated with increased genomic instability leading to the so-called mitotic catastrophe [20]. Finally, it has been shown that 14-3-3σ has a CpG-rich region (CpG island) within its first exon, which is hypermethylated in breast cancer [21], hepatocellular carcinoma [22], ovarial [23] and prostate cancer [24], with consecutive gene silencing. Together, 14-3-3σ appears to play an important role in primary tumor development. However, little is known about its possible contribution to tumor metastasis.

In the present report, the 14-3-3σ gene showed enhanced methylation in lymph node and cutaneous melanoma metastases compared with primary tumors, and gene methylation was associated with significant 14-3-3σ downregulation. Treatment of melanoma cells with methylation inhibitor 5-Aza-CdR and histone deacetylase inhibitor Pba led to a dramatic increase of 14-3-3σ expression associated with almost complete inhibition of cell proliferation. Similarly, 14-3-3σ overexpression resulted in cell cycle arrest and induction of melanoma cell senescence. These findings implicate an important role of 14-3-3σ in melanoma metastasis.

## Results

### 14-3-3σ mRNA expression is downregulated in melanoma metastases compared with primary melanomas

**Figure 1:**
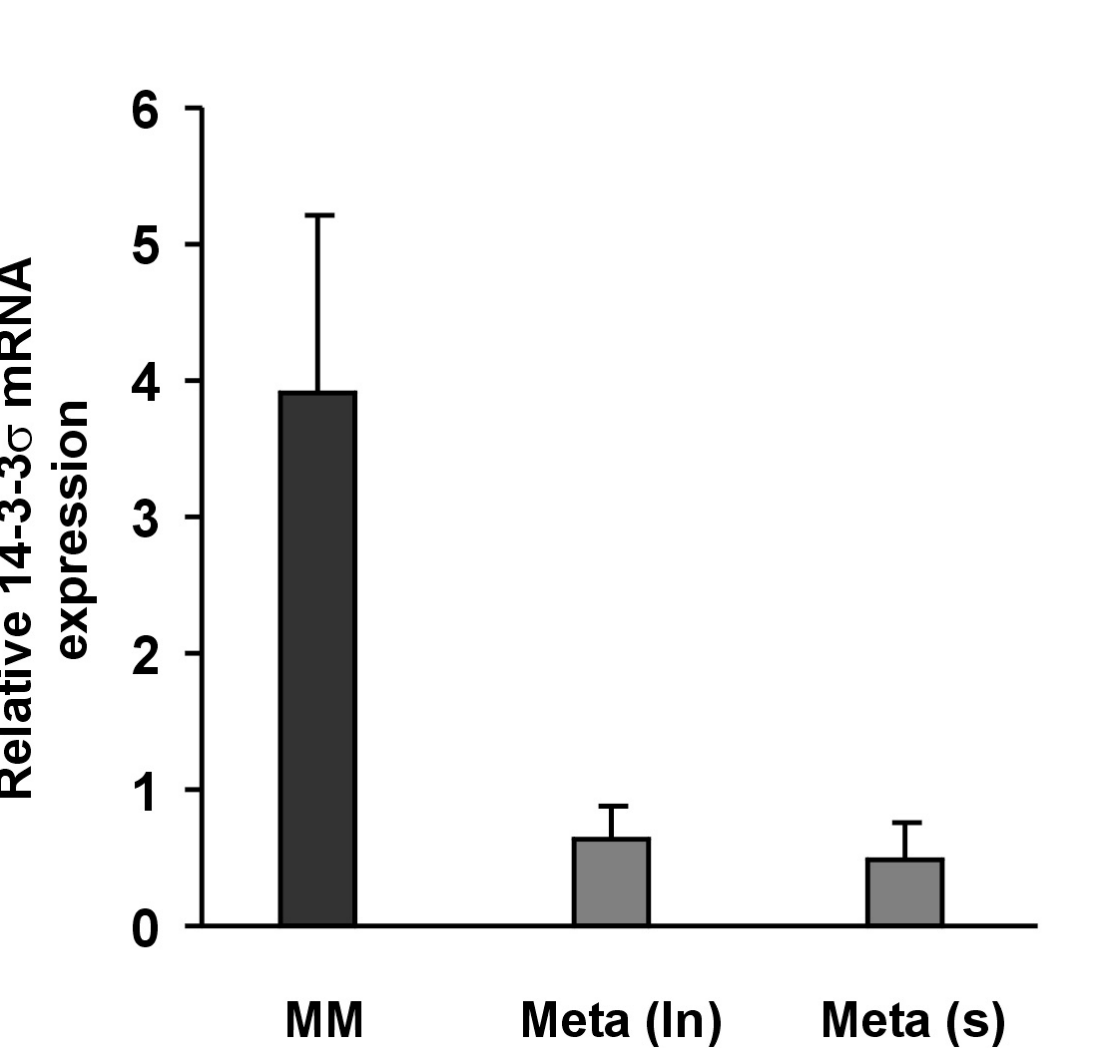
**14-3-3σ mRNA is downregulated in melanoma metastases compared with primary melanomas**. Total mRNA was extracted from melanoma cells excised by laser-capture microdissection from primary melanomas (n = 11), lymph node (n = 10) and cutaneous metastases (n = 12), respectively. Quantitative *real-time *PCR was performed and relative mRNA expression levels are given as means +/- S.E.M. MM, primary melanomas; Meta (ln), lymph node metastases; Meta (s), cutaneous metastases.

Previously, we were able to show in a large-scale gene expression study that 14-3-3σ was the most significantly downregulated gene in cutaneous melanoma metastases compared with primary melanomas [25]. In order to further substantiate and extend these findings, quantitative *real-time *PCR was performed for 14-3-3σ gene expression in an independent set of laser-microdissected primary melanomas, lymph node and cutaneous metastases. As shown in Figure 1, mean 14-3-3σ gene expression levels were close to 10-fold higher in primary tumors compared with metastases. Expression levels in both types of metastases, lymph node (ln) and cutaneous (s) metastases, were very similar. Taken together, melanoma progression appears to be associated with a significant downregulation of 14-3-3σ expression.

### The 14-3-3σ gene shows enhanced methylation in melanoma metastases compared with primary melanomas

**Figure 2:**
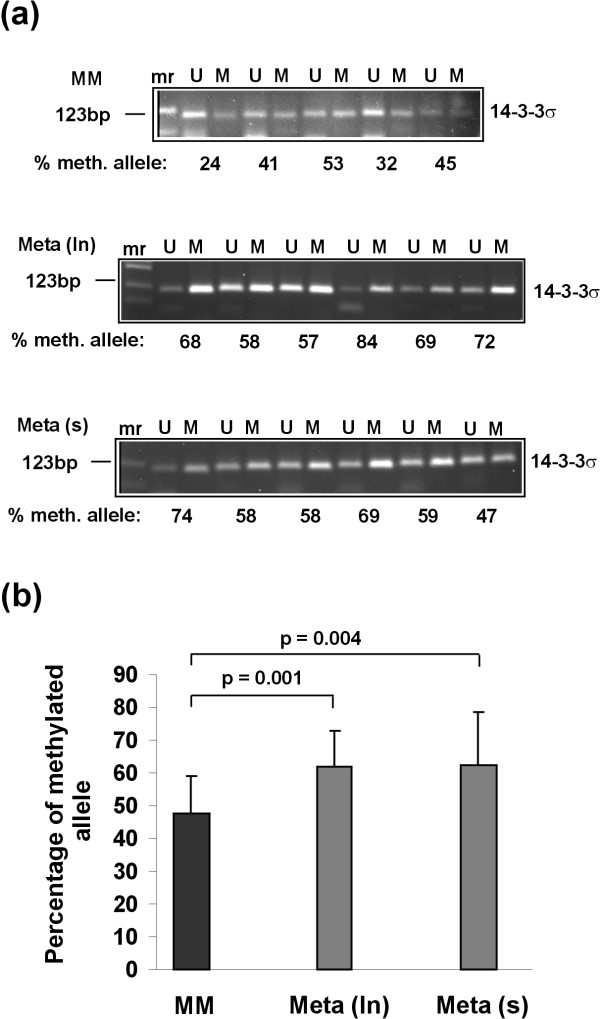
**The 14-3-3σ gene shows enhanced methylation in melanoma metastases compared with primary melanomas**. Methylation of the 14-3-3σ gene was analyzed by methylation-specific PCR. For this purpose genomic DNA extracted from laser-microdissected melanoma tissues was bisulfite-treated and subjected to PCR amplification of the 14-3-3σ gene with primers specific for either unmethylated (U) or methylated (M) DNA alleles. (**a**) The percentage of methylated fraction is given for a representative set of tumors in each group after densitometric quantification of the PCR products separated by agarose gel electrophoresis. (**b**) Summary of the percentages of methylated alleles of all samples tested (15 primary melanomas, 20 lymph node metastases and 19 cutaneous metastases). Data are given as means +/- SD, *P *values represent statistical significance after applying Student's *t-test*. MM, primary melanomas; Meta (ln), lymph node metastases; Meta (s), cutaneous metastases.

Epigenetic silencing of tumor suppressor genes is a common finding in primary malignancies [26]. As mentioned above, 14-3-3σ has been shown to be silenced in a series of primary tumors via gene methylation. However, little is known about silencing of 14-3-3σ gene during tumor metastasis. Here, 14-3-3σ gene methylation was analyzed in two different tumor tissues representing locoregional (lymph node) and distant (cutaneous) metastases, using methylation-specific PCR (MSP). Primers were spanning the region between CpG dinucleotides 3 and 9 of the first exon of the 14-3-3σ gene, commonly methylated in a variety of tumors. Representative PCR products generated by MSP and separated by agarose gels are shown in Figure 2a. All tumors tested were methylated at these CpG dinucleotides, but to a different extent. The percentage of methylated alleles in primary tumors was significantly lower than that of both metastasis groups. Figure 2b summarizes data obtained from 15 primary melanomas, 20 lymph node (ln) metastases and 19 cutaneous (s) metastases, respectively. Taken together, the level of CpG methylation of the 14-3-3σ gene in malignant melanoma increases during tumor progression. 14-3-3σ gene methylation might contribute to 14-3-3σ gene downregulation during melanoma metastasis.

### Treatment of melanoma cells with 5-Aza-CdR and Pba induces 14-3-3σ expression in melanoma cell lines

**Figure 3:**
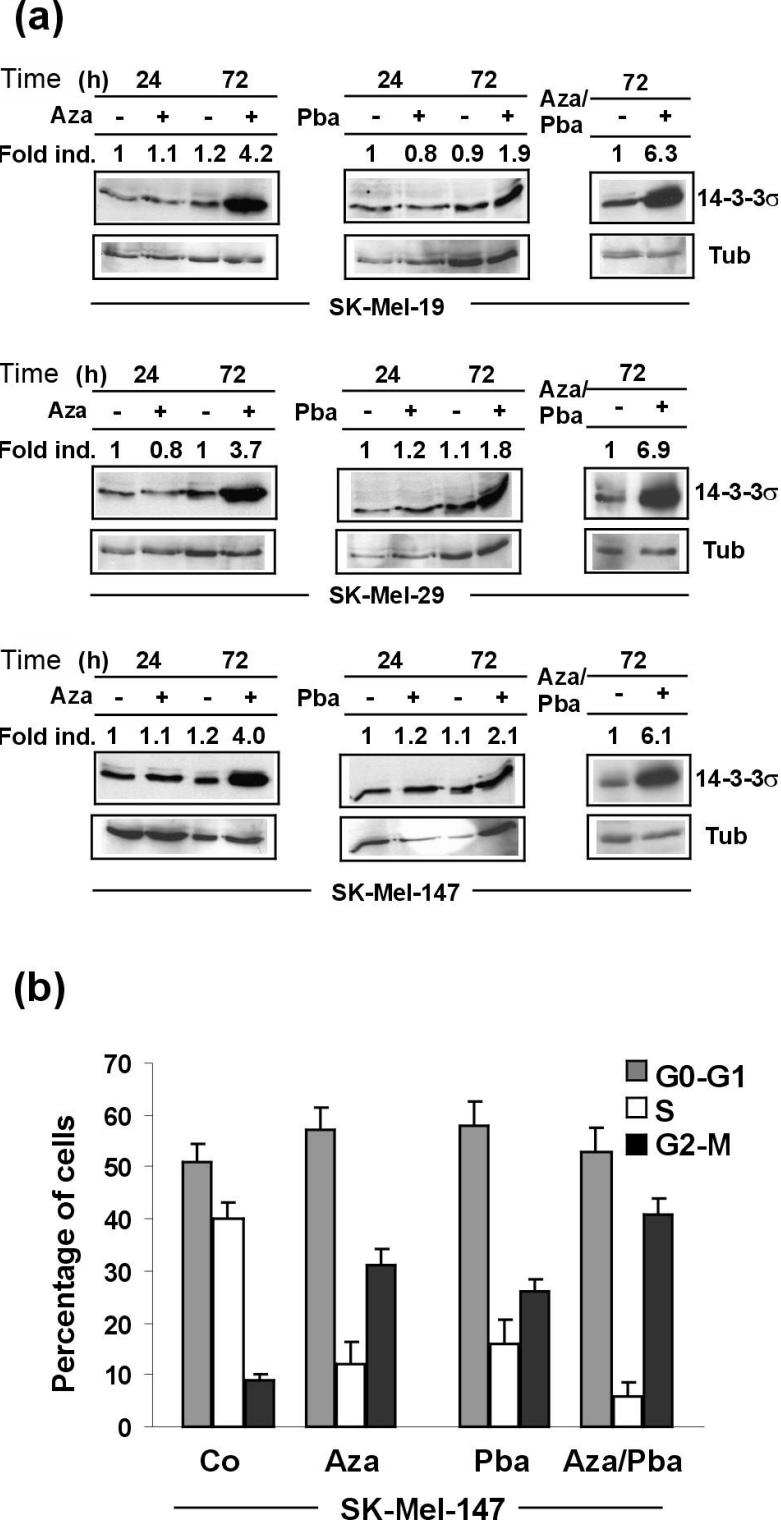
**Treatment of melanoma cells with 5-Aza-CdR, Pba, and 5-Aza-CdR/Pba, respectively, induces 14-3-3σ expression and leads to inhibition of cell cycle progression**. (**a**) 14-3-3σ immunoblot analyses of melanoma cell lines SK-Mel-19, SK-Mel-29 and SK-Mel-147, treated with 5-Aza-CdR (3 μM), Pba (3 mM), and a combination of both, respectively, for indicated times points or left untreated. (**b**) Cell cycle analysis of melanoma cell line SK-Mel-147 by flow cytometry after treatment as described in (a). Data are given as mean values +/- SD of four independent experiments.

To further substantiate the notion that downregulation of the 14-3-3σ gene in melanoma cells might be due to epigenetic silencing by gene hypermethylation, metastatic cell lines SK-Mel-19, SK-Mel-29, and SK-Mel-147, all showing more than 50% methylated 14-3-3σ alleles (data not shown), were treated with DNA methyltransferase inhibitor 5-Aza-CdR, deacetylase inhibitor Pba, and a combination of both, respectively. As shown in Figure 3a, treatment with both substances significantly induced 14-3-3σ protein expression, with 5-Aza-CdR having a significantly greater effect than Pba. Similar results were obtained in all three cell lines, indicating that epigenetic silencing of 14-3-3σ via gene methylation is a general phenomenon in melanoma cells. Combined treatment with both substances further increased 14-3-3σ expression rates. In summary, these findings showed that 14-3-3σ expression can dramatically be induced by demethylating agents such as 5-Aza-CdR. Combined treatment of 5-Aza-CdR with Pba further increased 14-3-3σ expression, which might be due to the fact that methylation and deacetylation act cooperatively during gene silencing.

### Induction of 14-3-3σ by 5-Aza-CdR and Pba induces cell cycle arrest in G0-G1 and G2-M phase of cell cycle

14-3-3σ is a major cell cycle checkpoint molecule inhibiting uncontrolled mitosis and proliferation of cells after DNA damaging stresses, with consecutive G2-M and (to a lesser extent) G0-G1 arrest. To address the question whether induction of 14-3-3σ expression after demethylation/inhibition of histone deacetylation impacts on cell cycle regulation in melanoma cells, cell cycle analyses were performed. SK-Mel-147 melanoma cells were treated with 5-Aza-CdR, Pba, and a combination of both, respectively, stained with BrdU and analyzed by flow cytometry. As shown in Figure 3b and Additional File 1, 5-Aza-CdR induced 14-3-3σ expression and dramatically reduced the number of cells in S-phase, paralleled by a slightly increased number of cells in G0-G1 and a significantly increased number of cells in G2-M. Pba alone had less dramatic effects. A combination of both further reduced the number of cells in S-phase and increased the number of cells in G2-M, compared with 5-Aza-CdR alone.

**Figure 4:**
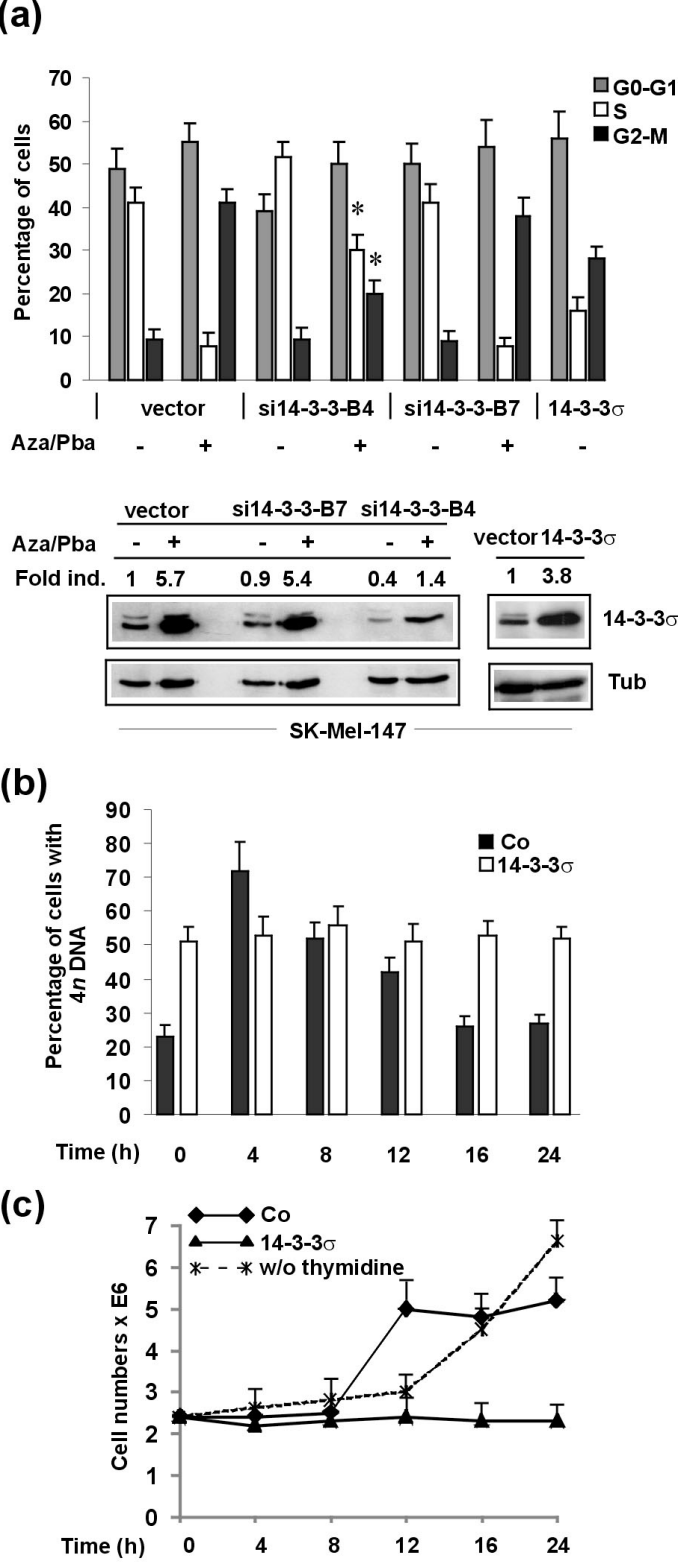
**14-3-3σ significantly contributes to 5-Aza-CdR/Pba-induced cell cycle inhibition of melanoma cells**. (**a**) Cell cycle analyses were performed for SK-Mel-147 melanoma cells transduced with empty vector, a functional (si14-3-3B4) and non-functional (si14-3-3B7) shRNA, respectively, treated with a combination of 5-Aza-CdR (3 μM) and Pba (3 mM) and (upper panel). 14-3-3σ stably ovexpressing cells after lentiviral transduction served as positive control. Data are given as mean values +/- SD of four independent experiments. Asterisks indicate statistical significance of differences in the number of cells in S or G2-M between vector and si14-3-3σ transduced cells (*P *< 0.05; Student's *t-test*). Lower panel shows respective immunoblots. (**b**) Time course of cell cycle progression of synchronized SK-Mel-147 melanoma cells after release of double thymidine block. Data are given as mean values +/- SD of four independent experiments. (**c**)Cell numbers of (b) at different time points after release of double thymidine block, together with cell numbers from an untreated control culture of SK-Mel-147 melanoma cells.

To further analyze the specific contribution of 14-3-3σ to 5-Aza-CdR/Pba-induced cell cycle arrest, melanoma cells were stably transduced with 14-3-3σ shRNA and 14-3-3σ cDNA. In preliminary experiments, a series of 7 different shRNAs have been tested (data not shown) and the one with the most significant gene knockdown (si14-3-3-B4) was used in cell cycle experiments together with a shRNA with no downmodulating effect on 14-3-3σ (si14-3-3-B7). As demonstrated in Figure 4a (upper panel), the inhibitory effects of 5-Aza-CdR/Pba treatment on cell proliferation (cells in S-phase) could partly be reversed in 14-3-3σ knockdown cells. However, complete reversion was not achieved, most likely be due to the fact that other 5-Aza-CdR/Pba-induced mechanisms might contribute to inhibition of melanoma cell proliferation. In line with these findings, 14-3-3σ stably overexpressing SK-Mel-147 cells (indicated as 14-3-3σ) showed a dramatic reduction of the fraction of proliferating cells (cells in S-phase). Figure 4a (lower panel) shows corresponding immunoblots, indicating that stably shRNAB4 transduced melanoma cells showed protein expression similar to that of unstimulated melanoma cells, while 14-3-3σ cDNA transduced cells showed significant upregulation of the 14-3-3σ protein. Taken together, these findings show that 14-3-3σ might be an important regulator of melanoma cell growth via inhibition of cell cycle progression, and loss of its expression might lead to uncontrolled growth of metastases.

To further substantiate the findings of an inhibitory activity of 14-3-3σ on cell cycle progression of melanoma cells, time course experiments were performed. Synchronous 14-3-3σ overexpressing SK-Mel-147 melanoma cells or control empty vector transduced melanoma cells were analyzed for cell cycle progression after release of double thymidine block (Figure 4b and Additional File 2). In these experiments, the number of cells with 4*n *DNA content (diploid cells) was analyzed at indicated time points. In contrast to empty vector control cells, which normally progressed through cell cycle, cells stably overexpressing 14-3-3σ showed a stable population of 4*n *DNA containing cells for the 24 h period analyzed. Cell numbers were counted at each time point and synchronization could be demonstrated for control cells, which doubled 8 h after release of thymidine block within 4 h. As expected, the numbers of 14-3-3σ overexpressing, G2-M arrested cells did not change. Untreated cells showed constant growth with transition to exponential growth in the late phase of the 24 h period. In summary, 14-3-3σ significantly impacts on cell cycle progression in melanoma cells, predominantly by arresting cells in G2-M phase.

### 14-3-3σ is involved in cellular senescence of melanoma cells

**Figure 5:**
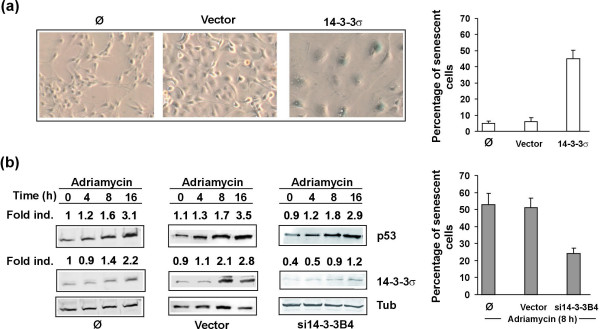
**14-3-3σ induces cellular senescence in melanoma cells**. (**a**) Expression of senescence marker SA-β-Gal in untransduced, vector control or 14-3-3σ cDNA transduced SK-Mel-147 melanoma cells. (**b**) SK-Mel-19 melanoma cells were exposed to 3.0 μg/ml adriamycin for indicated time points to induce cellular senescence. Induction of p53, 14-3-3σ and SA-β-Gal was analyzed by immunoblotting or immunohistochemistry in untransduced, vector control or 14-3-3σ shRNA (si14-3-3B4) transduced cells.

The fact the oncogene-induced senescence is dependent on intact cell cycle control via tumor suppressor genes directly interfering with cell cycle such as p16^INK^, prompted us to test the possibility that 14-3-3σ might be involved in melanoma cell senescence. In a first series of experiments, 14-3-3σ overexpressing and control melanoma cells were analyzed for senescence-associated β-galactosidase (SA-β-Gal) activity, a common marker for cellular senescence [27]. As shown in Figure 5a, 14-3-3σ overexpressing cells showed a high proportion of senescent cells compared with normal untransduced and empty vector transduced cells. In order to study the possible contribution of 14-3-3σ to cellular senescence induced by genotoxic stress, melanoma cells were treated with chemotherapeutic agent adriamycin. Indeed, adriamycin induced p53 and 14-3-3σ in control untransduced and vector transduced cells which resulted in a significant number of SA-β-Gal positive cells (Figure 5b). Interestingly, knockdown cells showed a considerably lower number of senescent cells (Figure 5b). Together, these findings indicate that 14-3-3σ is an inducer of cellular senescence of melanoma cells and is involved in the genotoxic stress-induced senescence program of these cells. Thus, loss of 14-3-3σ and normal senescence program might contribute to melanoma progression.

### Inhibition of 14-3-3σ induces enhanced migration of melanoma cells

**Figure 6:**
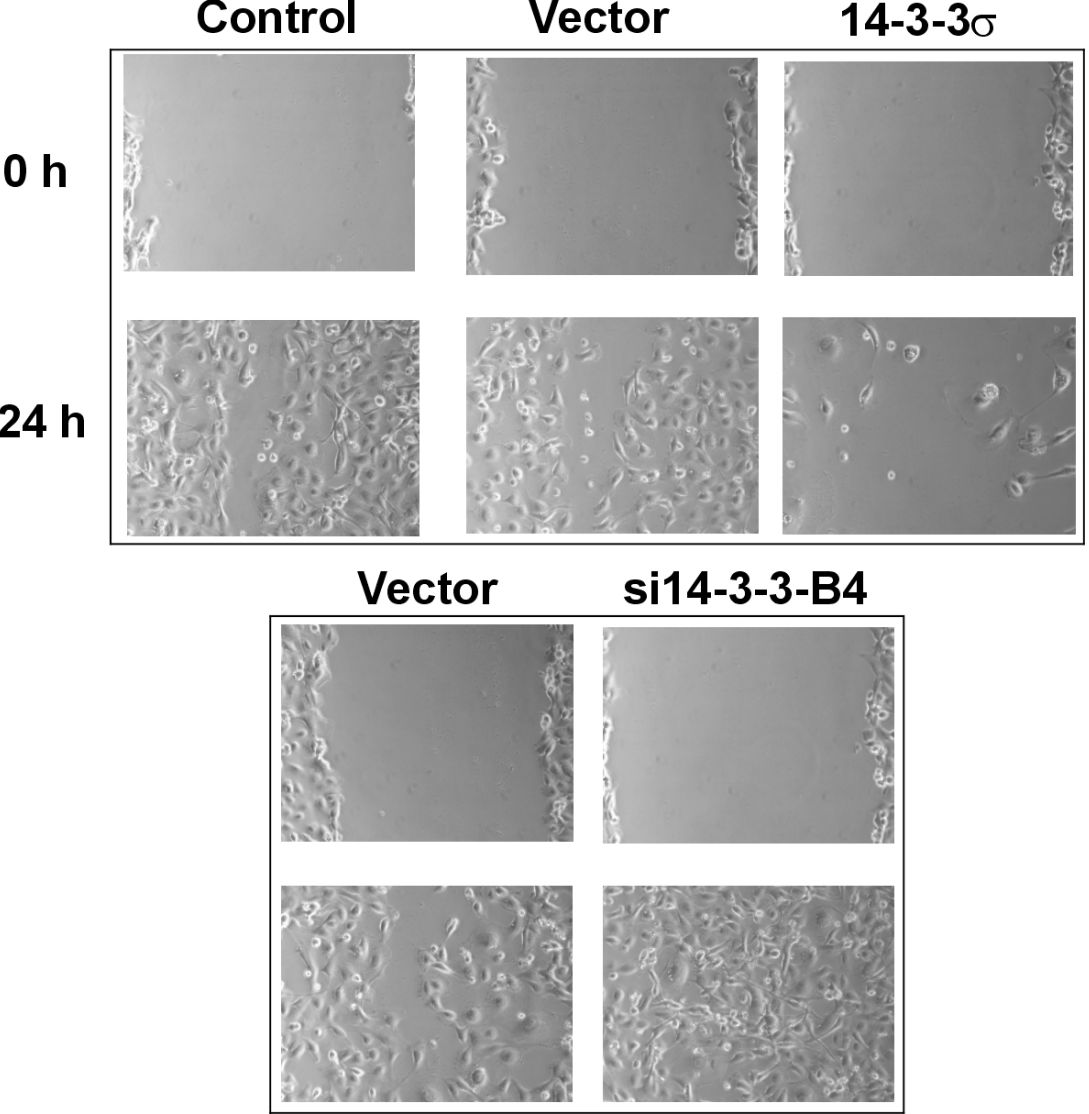
**14-3-3σ negatively interferes with melanoma cell migration**. Stably transfected SK-Mel-147 melanoma cells with respective empty control vector, 14-3-3σ cDNA and 14-3-3σ shRNA (si14-3-3B4), respectively, were used to test the influence of 14-3-3σ on melanoma cell migration. A wounding assay was performed and wound closure was analyzed after 24 h. For inhibition of cell proliferation 10 μg/ml mitomycin C was added 3 h before wounding of the cell layer.

Finally, we addressed the question whether 14-3-3σ interferes with melanoma cell migration as shown earlier for colon carcinoma cells [16]. Enhanced migratory capacity is a particular feature of metastasizing melanoma cells. For this purpose, a wounding assay was performed. In this assay cell proliferation was inhibited by addition of mitomycin C. As shown in Figure 6, normal control SK-Mel-147 melanoma cells showed almost complete closure of a wound in a confluent monolayer after 24 h, whereas 14-3-3σ overexpression totally inhibited wound closure. Even after 72 h there was only very limited number of tumor cells in the wound (data not shown). In contrast, 14-3-3σ knockdown cells showed complete closure of the wound after 24 h. 14-3-3σ overexpressing cells displayed a more enlarged and flattened morphology, which had been described earlier for colon carcinoma cells [15]. Taken together, 14-3-3σ significantly impacts on cellular migration of melanoma cells. Down-modulation of 14-3-3σ in melanoma metastases might thereby contribute to enhanced migratory capacity of metastatic melanoma cells.

## Discussion

In an earlier large-scale microarray study from our group, 14-3-3σ showed the most significant downregulation in metastatic melanoma lesions compared with primary tumors [25]. In the present report, we reconfirmed and extended these findings in an independent set of specimens, and further addressed the question of the underlying molecular mechanisms and the putative role of 14-3-3σ in the biology of melanoma metastasis.

Here, we demonstrated that downregulation of 14-3-3σ expression in metastatic melanoma lesions correlated with enhanced 14-3-3σ gene methylation. 14-3-3σ gene methylation indeed appeared to mediate gene silencing since *in vitro *experiments showed that treatment of melanoma cells with a methylated 14-3-3σ gene with demethylating agent 5-Aza-CdR (and histone deacetylation inhibitor Pba) led to dramatic up-regulation of 14-3-3σ in melanoma cells. In further experiments, 14-3-3σ overexpressing melanoma cells after 5-Aza-CdR/Pba treatment showed dramatically reduced proliferation rates, with cells predominantly arrested in G2-M. Furthermore, 14-3-3σ overexpression induced cellular senescence and was involved in genotoxic stress-induced senescence in melanoma cells. Moreover, we demonstrated that 14-3-3σ overexpression significantly reduced migratory capacity of melanoma cells.

Hypermethylation of the 14-3-3σ gene occurs at a CpG-rich region (CpG island) within its first exon [21], and there are series of different malignant tumors with a hypermethylated 14-3-3σ gene [21-24]. In the study by Ferguson and co-workers, gene methylation was associated with reduced 14-3-3σ expression, as 14-3-3σ-negative breast cancer cell lines were fully methylated at all of the 27 CpG dinucleotides of this exon [21]. Moreover, in the mentioned study ten 14-3-3σ-negative primary breast tumors showed partial or complete methylation of the 27 CpG dinucleotides. Overall, the strong correlation between methylation status and gene expression suggests that 14-3-3σ gene expression in primary tumors is significantly influenced by epigenetic mechanisms. In a subsequent study, Umbricht and co-workers demonstrated that 14-3-3σ is indeed an early event in breast cancer development [28]. Authors found hypermethylation of 14-3-3σ in 24 of 25 carcinomas, 15 of 18 ductal carcinomas *in situ*, and three of eight of atypical hyperplasias. In contrast, none of the benign hyperplasias without atypia showed 14-3-3σ hypermethylation. Interestingly, in patients with preinvasive and invasive breast cancer hypermethylation extended beyond the margins of the breast cancer tissue and involved normal breast tissue. The notion of early silencing of 14-3-3σ during tumor development was supported by experiments showing that immortalization of primary epidermal keratinocytes could be achieved only by 14-3-3σ downmodulation using 14-3-3σ antisense constructs, without the need of oncogenes or oncoviruses [29].

Interestingly, none of the currently available studies has addressed the role of 14-3-3σ in metastatic tumor growth. Here, we showed that 14-3-3σ was dramatically downregulated in metastatic lesions from lymph nodes and skin. Both represent different stages of metastatic spread, i.e., loco-regional (lymph node) and distant (skin) metastasis. In line with the above mentioned findings in primary tumors, 14-3-3σ downmodulation correlated with levels of gene methylation. Together, as derived from these findings, 14-3-3σ epigenetic silencing appears to be intensified as the tumors further progress towards metastasis. Interestingly, however, levels of gene expression and gene methylation did not differ between different stages/localizations of metastases. It should be mentioned that it is not conclusive from the present study whether the epigenetic regulation of 14-3-3σ is directly responsible for the observed gene silencing effect. Further experimentation is required to address this issue.

14-3-3 proteins, and in particular 14-3-3σ, play a central role in cell cycle regulation at different cell cycle checkpoints [11,12]. Blocking of 14-3-3σ activity by phosphoserine peptides containing a conserved 14-3-3 binding motif led to premature entry into mitosis and so-called mitotic catastrophe [30]. One of the major mechanisms by which 14-3-3 proteins exert checkpoint control is by sequestration of 14-3-3-interacting cell cycle molecules from the nucleus into cytoplasm. 14-3-3 interacting proteins are for example CDC25C and CDC25B, dual-specificity phosphatases and activators of cyclin-dependent kinase CDC2, the central kinase for driving cells through mitosis. During interphase, premature activation of CDC2 is prevented by inactivation of both CDC25 proteins by 14-3-3-mediated cytoplasmic sequestration. 14-3-3σ, however, does not directly interact with CDC25C, but appears to negatively regulate mitosis by sequestration of CDC2-cyclin B complexes [31]. The exact functional role of 14-3-3σ binding to CDC25B remains to be determined. 14-3-3σ also binds to cyclin dependent kinases CDK2 and CDK4, both of which are active during G1, and might thereby contribute to G1 arrest [32]. Moreover, 14-3-3 proteins bind to and inactivate CDC25A by cytoplasmic sequestration. CDC25A dephosphorylates CDK2 on its inhibitory phosphates and thereby promotes S-phase entry. Binding to CDC25A has, however, not yet been shown for 14-3-3σ. Together, the major effects of 14-3-σ appear to be in G2-M, but entry into cell cycle (G0-G1) might also be affected [12,15].

By use of 5-Aza-CdR alone or in a combination with Pba, we induced high levels of 14-3-3σ in melanoma cells *in vitro*. Parallel cell cycle analyses by flow cytometry showed that the number of cells in S-phase was dramatically reduced, with a shift of cells to G2-M and to a lesser extend to G0-G1. Similar results were obtained in 14-3-3σ stably overexpressing cells. Together, these findings indicated that 14-3-3σ leads to cell cycle arrest of melanoma cells mainly in G2-M. In line with this, synchronized 14-3-3σ overexpressing cells showed a invariable number of 4*n *DNA containing cells in time course experiments, while control cells normally progressed through cell cycle. Similar findings have been reported earlier in colon carcinoma cells [15].

Interestingly, recently published data indicated that 14-3-3σ also plays role in mitotic exit [33]. Authors demonstrated a previously unknown function for 14-3-3σ as a regulator of mitotic translation through its direct mitosis-specific binding to a variety of translation/initiation factors. Cells lacking 14-3-3σ could not suppress cap-dependent translation and did not stimulate cap-independent translation during and immediately after mitosis. This defective switch in the mechanism of translation resulted in impaired cytokinesis and the accumulation of binucleate cells. This was regarded as a potential explanation of how 14-3-3σ-deficient cells progress on their way to aneuploidy, a characteristic feature of malignant transformation. The fact that 14-3-3σ is further downregulated in metastatic tumor cells, as exemplified by our study, might explain high levels of aneuploidy in metastatic tumor cells [34,35].

In a further series of experiments, we addressed the question, whether 14-3-3σ might be involved in regulation of cellular senescence of melanoma cells, as cellular senescence is tightly linked to cell cycle control and activation of tumor suppressor p53 in many cell types [36]. SA-β-Gal activity is strongly expressed in benign melanocytic nevi, the common precursors of malignant melanoma [37]. However, it remains to be determined whether the normal cellular senescence program might be compromised in primary or metastatic melanoma lesions. The p19^ARF^/p53- and Rb/p16^INK4a^-dependent pathways mediate cellular senescence after oncogene stimulation in a variety of premalignant and malignant tumors including malignant melanoma [36,38]. However, evidence has been provided that an Rb/p16 independent mechanism via inactivation of T-box transcription factor Tbx2, which is overexpressed in melanomas, can also lead to melanoma cell senescence [39]. This mechanism appears may involve p53 and p21^Cip1^, as Tbx2 and Tbx3 are potent repressors of p21^Cip1^. Since p53 is upregulated in a positive feedback loop via 14-3-3σmediated inhibition of p53 ubiquitinating HDM2 [17,18], overexpression of 14-3-3σ might overcome this senescence block in melanoma cells, or downregulation of 14-3-3σ might be supportive for defective senescence program. Indeed, we demonstrated that 14-3-3σ overexpression induced cellular senescence *in vitro*. Moreover, stress-induced senescence by chemotherapeutic agent adriamycin was at least in part dependent on 14-3-3σ expression, as 14-3-3σ knockdown cells showed significantly less senescent cells. Thus, 14-3-3σ may overcome defective senescence programs in melanoma cells. In line with recent reports showing that cellular senescence of human melanocytes involved histone deacetylase 1 (HDAC1) [40,41], which deacetylates histone lysine residues as a major prerequisite for histone H3K9 methylation, we found significant H3K9 methylation in adriamycin-induced 14-3-3σ mediated senescence (data not shown).

A more direct role of 14-3-3σ in cellular senescence was shown earlier [29]. Authors showed that 14-3-3σ is abundantly expressed in the suprabasal differentiated layers of human epidermis. Moreover, its downregulation allows keratinocytes to escape replicative senescence by impairing clonal evolution. Downregulation of 14-3-3σ was accompanied by the maintenance of telomerase activity and by a strong downregulation of the *p16*^INK4a ^tumor suppressor gene. Together, based on our findings and the mentioned data from other groups, loss of a functional senescence pathway via 14-3-3σ downmodulation might be a further mechanism for melanoma metastasis *in vivo*.

Finally, we tested the capacity of 14-3-3σ to interfere with cellular migration of melanoma cells. It is well understood that enhanced migratory capacity is a characteristic of metastatic tumor cells [35,42]. In a recent study using tandem affinity purification and the multidimensional protein identification technology 117 proteins were identified as interaction partners for 14-3-3σ in human cells [16]. Many of these were involved in mitogenic signalling and cell cycle. However, the largest functional class was represented by 36 proteins involved in cellular migration, including RhoE, RhoGEF16 and RhoGEF17, Rac GTPase-activating protein 1, C-terminal tensin-like and ladinin. In line with this, we observed strong migratory inhibitory effects on melanoma cells in a wounding assay. Thus, downregulated 14-3-3σ in melanoma cells might lead to increased motility and further support tumor metastasis. Similar results were reported for the 14-3-3σ overexpressing DLD1 colon cancer cell line [16].

## Conclusion

In summary, epigenetic silencing of 14-3-3σ in melanoma metastases might contribute to tumor progression via loss of cell cycle control and control of cellular senescence programs as well as by support of melanoma cell migratory capacity.

## Methods

### Melanoma tissues and cell lines

For quantitative real-time PCR (qPCR) and methylation-specific PCR (MSP) two independent sets of melanoma specimens were used. The first set (qPCR) consisted of 11 primary melanomas, 10 lymph node metastases and 12 cutaneous metastases, respectively. The second set (MSP) consisted of 15 primary melanomas, 20 lymph node metastases and 19 cutaneous metastases. More detailed information about primary melanomas including tumor subtype, tumor thickness and clinical stage is given in Additional Files 3 and 4. These studies were performed according to the Principles of the Declaration of Helsinki and were approved by the local Ethics Committee at the University of Rostock. Biopsies were obtained from patients after informed consent. The human melanoma cell lines SK-Mel-19, SK-Mel-29, and SK-Mel-147 were kindly provided by M. Soengas, Department of Dermatology, University of Michigan, Ann Arbor, MI, U.S.A. [38]. Cell cultures were maintained in DMEM medium supplemented with 10% fetal calf serum and 100 μg penicillin-streptomycin ml^-1^.

### RNA purification and real-time RT-PCR

For 14-3-3σ mRNA expression analysis in primary melanomas and metastases laser-capture microdissection of tissues was performed as described earlier [25]. Total RNA was extracted from laser-microdissected melanoma cells, using a commercially available RNA extraction Kit (RNeasy^®^, Qiagen, Hilden, Germany). RNA concentrations were determined with a NanoDrop^® ^ND-1000 spectrophotometer (NanoDrop Technologies, Wilmington, DE, USA), and RNA quality was tested with an Agilent 2100 Bioanalyzer (Agilent Technologies, Santa Clara, CA, USA). A commercially available TaqMan^® ^*real-time *PCR assay was performed (Assays-on-Demand^®^, Applied Biosystems, Darmstadt, Germany). Ten ng of total RNA were used in each reaction and reverse transcription of RNA templates and PCR amplification were carried out with the TaqMan EZ RT-PCR^® ^Kit using an ABI PRISM^® ^7700 system (Applied Biosystems). Data were normalized to GAPDH expression.

### Methylation-specific PCR (MSP)

Genomic DNA was extracted from laser-microdissected tissues from primary melanomas and melanoma metastases using DNeasy™ Tissue Kit (Qiagen). One μg of DNA was treated with sodium bisulfite using CpGenome™ Fast DNA Modification Kit (Chemicon, Hofheim, Germany). Subsequently, methylation-specific PCR (MSP) was performed with a primer set that covered CpG dinucleotide numbers 3, 4, 8 and 9 [21]. Primers specific for methylated DNA had the sequence: 5'-TGGTAGTTTTTATGAAAGGCGTC-3' (sense) and 5'-CCTCTAACCGCCCACCACG-3' (antisense), primers specific for unmethylated DNA had the sequence: 5'-ATGGTAGTTTTTATGAAAGGTGTT-3' (sense) and 5'-CCCTCTAACCACCCACCACA-3' (antisense). Sizes of amplification products were 105 bp and 107 bp, respectively. The PCR conditions were as follows: 1 cycle of 95°C for 5 min; 30 cycles of 95°C for 45 s, 56°C for 30 s and 72°C for 30 s; and 1 final cycle of 72°C for 10 min. PCR products were separated on 1% agarose gels and stained with ethidium bromide. For quantification of PCR products the volumes of the different bands of the gel were analyzed using Progenesis PG200 software (Nonlinear Dynamics, Newcastle, UK). Experimental results were expressed as per cent methylated band signal.

### 5-Aza-CdR and Pba treatment of cell lines

SK-Mel-19, SK-Mel-29 and SK-Mel-147 melanoma cells (1.5 × 10^6^) were treated with 5-Aza-CdR (3 μM; Sigma-Aldrich, Munich, Germany), Pba (3 mM; Sigma Aldrich), or a combination of both for 72 h or left untreated. 5-Aza-CdR was removed after 24 h, while Pba was administered continuously during the 72 h period by replacement of medium.

### Antibodies and immunoblotting

Melanoma cell lines were lysed on ice for 30 min using radioimmunoprecipitation (RIPA) buffer. Forty μg of total protein were denatured in electrophoresis sample buffer for 5 min at 95°C and subjected to SDS-polyacrylamide gel electrophoresis (PAGE). Gels were electroblotted onto nitrocellulose membranes (Highbond ECL^®^, Amersham, Braunschweig, Germany) and subjected to immunodetection. The following primary antibodies were used: anti-14-3-3σ mouse monoclonal antibody (ab14123, abcam/BIOZOL, Eching, Germany), anti-p53 mouse monoclonal antibody (cat. no. 554293, BD Biosciences; Heidelberg, Germany), anti-β-tubulin rabbit polyclonal antibody (sc-9104, Santa Cruz Biotechnology, Heidelberg, Germany). Signal detection was performed by appropriate secondary anti-mouse or anti-rabbit antibodies, coupled with horseradish peroxidase (Santa Cruz Biotechnology). A standard ECL (enhanced chemoluminescence) reaction (Amersham) was performed for signal visualization. Relative expression levels of 14-3-3σ and p53 in immunoblots were analyzed by ImageJ software and normalized to tubulin expression .

### Lentiviral 14-3-3σ shRNA or cDNA transduction

To establish SK-Mel-147 and SK-Mel-19 14-3-3σ knockdown cells, a vector-based shRNA technique was used. A set of seven different shRNAs cloned into pLKO.1 vector (RHS4533, Human pLKO.1 lentiviral shRNA target gene set; Open Biosystems, Huntsville, AL, U.S.A.) was used. Recombinant lentiviruses were produced by co-transfecting human embryonic kidney (HEK) 293 T cells with each 14-3-3σ shRNA lentiviral expression plasmid together with packaging plasmids pTLA1-Pak, pTLA1-Enz, pTLA1-Env, pTLA1-Rev and pTLA1-TOFF (Translentiviral shRNA Packaging System; Open Biosystems) using Effectene™ (Qiagen) as a transfection reagent. HEK 293 T cells were cultured in DMEM supplemented with 10% FCS, 100 μg penicillin-streptomycin ml^-1^, in a 37°C incubator with 5% CO_2_. Infectious lentiviruses were collected 48 h after transfection. The supernatant was centrifuged, filtered through 0.45 μm filters (Millipore, Carrigtwohill, Ireland) and concentrated by centrifugation with 50 000 g at 4°C for 90 min. After transduction melanoma cells were put in selection medium containing 1% puromycin. This medium was replaced by normal culture medium 24 h before start of experiments. 14-3-3σ gene knockdown was tested by quantitative PCR. The shRNA with the most significant (80%) knockdown, called si14-3-3-B4 (TRC0000040128; Open Biosystems), and one non-functional shRNA, called si14-3-3-B7 (TRC0000040131; Open Biosystems) were used in further functional experiments.

For stable transduction of SK-Mel-147 melanoma cells with 14-3-3σ cDNA, 14-3-3σ cDNA was cut out of a commercially available cDNA expression plasmid (pcDNA3.1/hisC14-3-3sigma; addgene, Germany) by restriction enzyme digest, using *Bam*HI and *Eco*RI restriction enzymes. The resulting fragment was cloned into the lentiviral expression vector pCDF1-MCS1-EF1-Puro (System Biosciences, Moutain View, CA, U.S.A.), which was used for stable transduction of melanoma cells. Infectious lentiviruses were produced using the pPACKF packaging system (System Biosciences). Again, after transduction melanoma cells were put in selection medium containing 1% puromycin. This medium was replaced by normal culture medium 24 h before start of experiments.

### Cell cycle analysis

For cell cycle analysis, SK-Mel-147 melanoma cells were stained using an FITC BrdU Flow Kit (BD Biosciences). Populations in G0-G1, S and G2-M phase were measured by flow cytometry with a FACSCalibur (BD Biosciences). Data were processed and analyzed by CellQuest™ Software (BD Biosciences). For time course experiments of cell cycle, SK-Mel-147 melanoma cells were subjected to double thymidine block (early S-phase block) for synchronization. For this purpose, cells were grown at 25–30% confluency, washed twice with 1 × PBS and subsequently grown in DMEM medium supplemented with 10% fetal calf serum and 100 μg penicillin-streptomycin ml^-1^and 2 mM thymidine (Sigma-Aldrich) for 18 h (first block). After that first block thymidine was removed by washing with 1 × PBS, fresh medium was added for 9 h to release cells. After releasing again medium with 2 mM thymidine was added for 17 h (second block). After the second block thymidine was removed by washing with 1 × PBS and cells were released by adding fresh DMEM medium. Cells then progress synchronously through G2 and mitotic phase. For measurement of *4n *DNA containing cells by flow cytometry, cells were stained with propidium iodide (Sigma-Aldrich). To confirm synchronization of melanoma cells, cell numbers were counted at each time point and compared with cell numbers of untreated cells from a parallel culture.

### Detection of cellular senescence

A commercially available system was used for detection of SA-β-Gal expression (Senescence Cell Histochemical Staining Kit, Sigma-Aldrich) and the staining of cells was performed as recommended by the manufacturer. SK-Mel-147 melanoma cells were stained with an X-gal containing staining mixture for 4 h at 37°C and blue-stained cells and total number of cells was counted by microscopic inspection.

### Wounding Assay

SK-Mel-147 melanoma cells were seeded into 6-well plates at 70–80% confluency. To prevent proliferation during wounding assay, cells were treated with 10 μg/ml mitomycin C (Sigma-Aldrich) for 3 h. The cell monolayer was then scratched in the middle with a plastic tip. Cell migration was monitored by optical inspection for 24 h using an Olympus microscope (Olympus, Hamburg, Germany) and pictures were taken at 0 and 24 h.

### Statistical analysis

Copy numbers of 14-3-3σ mRNA analyzed by quantitative *real-time *RT-PCR, the percentage of methylated 14-3-3σ alleles and percentage of senescent cells are given as mean +/- SD or +/- S.E.M, as indicated. For analysis of statistical significance after testing of normal distribution of values a Student's *t-test *was performed. Data with *P *≤ 0.05 were regarded as statistically significant.

## Abbreviations

The abbreviations used are: 5-Aza-CdR: 5'-aza-2'-deoxycytidine; Pba: 4-phenylbutyric acid; SA-β-Gal: senescence-associated β-galactosidase.

## Competing interests

The authors declare that they have no competing interests.

## Authors' contributions

JS carried out the molecular and epigenetic studies, cell cycle analysis, the wounding assays, lentiviral gene transduction, and senescence assays. SI participated in lentiviral gene transduction and cell cycle analysis. JV participated in the design of the study and performed the statistical analysis. MK conceived of the study, and participated in its design and coordination and helped to draft the manuscript. All authors read and approved the final manuscript.

## Supplementary Material

Additional File 1 **Treatment of melanoma cells with 5-Aza-CdR, Pba, and 5-Aza-CdR/Pba, respectively, leads to inhibition of cell cycle progression**. Cell cycle analysis of SK-Mel-147 melanoma cells was performed by measurement of BrdU uptake using flow cytometry after treatment of cells with 5-Aza-CdR, Pba, and 5-Aza-CdR/Pba, respectively. Flow cytometric profiles (dot blots) of one representative experiment are shown. Click here for file

Additional File 2 **Cell cycle progression of SK-Mel-147 cells after release of double thymidine bloc**. **(a) **Synchronized, control (empty vector) transduced SK-Mel-147 cells were released from double thymidine block and the number of *4n *DNA containing cells was analyzed by flow cytometry. **(b) **Synchronized, 14-3-3σ overexpressing SK-Mel-147 cells were released from double thymidine block and were analyzed as in (a). Histogram data from one representative experiment are shown in (a) and (b). Numbers in the diagrams indicate the percentages of *4n *DNA containing cells. Click here for file

Additional File 3 **Histopathological data and clinical stage of primary melanomas analyzed by quantitative real-time PCR for 14-3-3σ expression**. ^1) ^NMM, nodular malignant melanoma; SSM, superficial spreading malignant melanoma; LMM, lentigo maligna melanoma; ALM, acro-lentiginous malignant melanoma. ^2^) Classification of clinical stage was done according to Balch CM, Buzaid AC, Atkins MB, Cascinelli N, Coit DG, Fleming ID, Houghton Jr. A, Kirkwood JM, Mihm MF, Morton DL, Reintgen D, Ross MI, Sober A, Soong SJ, Thompson JA, Thompson JF, Gershenwald JE, McMasters KM. A new American Joint Committee on Cancer staging system for cutaneous melanoma. Cancer 2000;88: 1484–91. Click here for file

Additional File 4 **Histopathological data and clinical stage of primary melanomas analyzed by methylation-specific PCR**. Table showing histopathological data and clinical stage of primary melanomas analyzed by methylation-specific PCR. ^1)^/^2) ^Melanoma subtypes and classification of clinical stage are explained in text of Supplemental Table 1. Click here for file

## References

[B1] Miller AJ, Mihm MC (2006). Melanoma. N Engl J Med.

[B2] Chudnovsky Y, Khavari PA, Adams AE (2005). Melanoma genetics and the development of rational therapeutics. J Clin Invest.

[B3] Tucker MA, Goldstein AM (2003). Melanoma etiology: where are we?. Oncogene.

[B4] Chin L (2003). The genetics of malignant melanoma: lessons from mouse and man. Net Rev Cancer.

[B5] Thomas RK, Baker AC, Debiasi RM, Winckler W, Laframboise T, Lin WM, Wang M, Feng W, Zander T, MacConaill L, Lee JC, Nicoletti R, Hatton C, Goyette M, Girard L, Majmudar K, Ziaugra L, Wong KK, Gabriel S, Beroukhim R, Peyton M, Barretina J, Dutt A, Emery C, Greulich H, Shah K, Sasaki H, Gazdar A, Minna J, Armstrong SA, Mellinghoff IK, Hodi FS, Dranoff G, Mischel PS, Cloughesy TF, Nelson SF, Liau LM, Mertz K, Rubin MA, Moch H, Loda M, Catalona W, Fletcher J, Signoretti S, Kaye F, Anderson KC, Demetri GD, Dummer R, Wagner S, Herlyn M, Sellers WR, Meyerson M, Garraway LA (2007). High-throughput oncogene mutation profiling in human cancer. Nature Genet.

[B6] Davies H, Bignell GR, Cox C, Stephens P, Edkins S, Clegg S, Teague J, Woffendin H, Garnett MJ, Bottomley W, Davis N, Dicks E, Ewing R, Floyd Y, Gray K, Hall S, Hawes R, Hughes J, Kosmidou V, Menzies A, Mould C, Parker A, Stevens C, Watt S, Hooper S, Wilson R, Jayatilake H, Gusterson BA, Cooper C, Shipley J, Hargrave D, Pritchard-Jones K, Maitland N, Chenevix-Trench G, Riggins GJ, Bigner DD, Palmieri G, Cossu A, Flanagan A, Nicholson A, Ho JW, Leung SY, Yuen ST, Weber BL, Seigler HF, Darrow TL, Paterson H, Marais R, Marshall CJ, Wooster R, Stratton MR, Futreal PA (2002). Mutations of the BRAF gene in human cancer. Nature.

[B7] Maldonado JL, Fridlyand J, Patel H, Jain AN, Busam K, Kageshita T, Ono T, Albertson DG, Pinkel D, Bastian BC (2003). Determinants of BRAF mutations in primary melanomas. J Natl Cancer Inst.

[B8] Curtin JA, Fridlyand J, Kageshita T, Patel HN, Busam KJ, Kutzner H, Cho KH, Aiba S, Bröcker EB, LeBoit PE, Pinkel D, Bastian BC (2005). Distinct sets of genetic alterations in melanoma. New Engl J Med.

[B9] Dhillon AS, Kolch W (2004). Oncogenic B-Raf mutations: Cristal clear at last. Cancer Cell.

[B10] Pollock PM, Harper UL, Hansen KS, Yudt LM, Stark M, Robbins CM (2003). High frequency of BRAF mutations in nevi. Nat Genet.

[B11] Hermeking H (2003). The 14-3-3 cancer connection. Nat Rev Cancer.

[B12] Hermeking H, Benzinger A (2006). 14-3-3 proteins in cell cycle regulation. Semin Cancer Biol.

[B13] Muslin AJ, Tanner JW, Allen PM, Shaw AS (1996). Interaction of 14-3-3 with signaling proteins is mediated by the recognition of phosphoserine. Cell.

[B14] Tzivion G, Luo Z, Avruch J (1998). A dimeric 14-3-3 protein is an essential cofactor for Raf kinase activity. Nature.

[B15] Hermeking H, Lengauer C, Polyak K, He TC, Zhang L, Thiagalingam S, Kinzler KW, Vogelstein B (1997). 14-3-3 sigma is a p53-regulated inhibitor of G2/M progression. Mol Cell.

[B16] Benzinger A, Muster N, Koch HB, Yates JR, Hermeking H (2005). Targeted proteomic analysis of 14-3-3 sigma, a p53 effector commonly silenced in cancer. Mol Cell Proteomics.

[B17] Levine AJ (1997). p53, the cellular gatekeeper for growth and division. Cell.

[B18] Yang HY, Wen YY, Chen CH, Lozano G, Lee MH (2003). 14-3-3 sigma positively regulates p53 and suppresses tumor growth. Mol Cell Biol.

[B19] Yang HY, Wen YY, Lin YI, Pham L, Su CH, Yang H, Chen J, Lee MH (2007). Roles for negative cell regulator 14-3-3sigma in control of MDM2 activities. Oncogene.

[B20] Chan TA, Hermeking H, Lengauer C, Kinzler KW, Vogelstein B (1999). 14-3-3σ is required to prevent mitotic catastrophe after DNA damage. Nature.

[B21] Ferguson AT, Evron E, Umbricht CB, Pandita TK, Chan TA, Hermeking H, Marks JR, Lambers AR, Futreal PA, Stampfer MR, Sukumar S (2000). High frequency of hypermethylation at the 14-3-3 sigma locus leads to gene silencing in breast cancer. Proc Natl Acad Sci USA.

[B22] Iwata N, Yamamoto H, Sasaki S, Itoh F, Suzuki H, Kikuchi T, Kaneto H, Iku S, Ozeki I, Karino Y, Satoh T, Toyota J, Satoh M, Endo T, Imai K (2000). Frequent hypermethylation of CpG islands and loss of expression of the 14-3-3 sigma gene in human hepatocellular carcinoma. Oncogene.

[B23] Mhawech P, Benz A, Cerato C, Greloz V, Assaly M, Desmond JC, Koeffler HP, Lodygin D, Hermeking H, Herrmann F, Schwaller J (2005). Downregulation of 14-3-3sigma in ovary, prostate and endometrial carcinomas is associated with CpG island methylation. Mod Pathol.

[B24] Lodygin D, Diebold J, Hermeking H (2004). Prostate cancer is characterized by epigenetic silencing of 14-3-3sigma expression. Oncogene.

[B25] Jaeger J, Koczan D, Thiesen HJ, Ibrahim SM, Gross G, Spang R, Kunz M (2007). Gene expression signatures for tumor progression, tumor subtype and tumor thickness in laser-microdissected melanoma tissues. Clin Cancer Res.

[B26] Ting AH, McGarvey KM, Baylin SB (2006). The cancer epigenome–components and functional correlates. Genes Dev.

[B27] Dimri GP, Lee X, Basile G, Acosta M, Scott G, Roskelley C, Medrano EE, Linskensi M, Rubelj I, Pereira-Smith O, Peacocke M, Campisi J (1995). A biomarker that identifies senescent human cells in culture and in aging skin *in vivo*. Proc Natl Acad Sci USA.

[B28] Umbricht CB, Evron E, Gabrielson E, Ferguson A, Marks J, Sukumar S (2001). Hypermethylation of 14-3-3 sigma (stratifin) is an early event in breast cancer. Oncogene.

[B29] Dellambra E, Golisano O, Bondanza S, Siviero E, Lacal P, Molinari M, D'Atri S, De Luca M (2000). Downregulation of 14-3-3sigma prevents clonal evolution and leads to immortalization of primary human keratinocytes. J Cell Biol.

[B30] Nguyen A, Rothman DM, Stehn J, Imperiali B, Yaffe MB (2004). Caged phosphopeptides reveal a temporal role for 14-3-3 in G1 arrest and S-phase checkpoint function. Nat Biotechnol.

[B31] Dalal SN, Yaffe MB, DeCaprio JA (2004). 14-3-3 family members act coordinately to regulate mitotic progression. Cell Cycle.

[B32] Laronga C, Yang HY, Neal C, Lee MH (2000). Association of the cyclin-dependent kinases and 14-3-3 sigma negatively regulates cell cycle progression. J Biol Chem.

[B33] Wilker EW, van Vugt MA, Artim SA, Huang PH, Petersen CP, Reinhardt HC, Feng Y, Sharp PA, Sonenberg N, White FM, Yaffe MB (2007). 14-3-3σ controls mitotic translation to facilitate cytokinesis. Nature.

[B34] Lengauer C, Kinzler KW, Vogelstein B (1998). Genetic instabilities in human cancers. Nature.

[B35] Hanahan D, Weinberg RA (2000). The hallmarks of cancer. Cell.

[B36] Campisi J (2005). Senescent cells, tumor suppression, and organismal aging: good citizens, bad neighbors. Cell.

[B37] Michaloglou C, Vredeveld LC, Soengas MS, Denoyelle C, Kuilman T, Horst CM van der, Majoor DM, Shay JW, Mooi WJ, Peeper DS (2005). BRAFE600-associated senescence-like cell cycle arrest of human naevi. Nature.

[B38] Soengas MS, Capodieci P, Polsky D, Mora J, Esteller M, Opitz-Araya X, McCombie R, Herman JG, Gerald WL, Lazebnik YA, Cordón-Cardó C, Lowe SW (2001). Inactivation of the apoptosis effector Apaf-1 in malignant melanoma. Nature.

[B39] Vance KW, Carreira S, Brosch G, Goding CR (2005). Tbx2 is overexpressed and plays an important role in maintaining proliferation and suppression of senescence in melanomas. Cancer Res.

[B40] Bandyopadhyay D, Okan NA, Bales E, Nascimento L, Cole PA, Medrano EE (2002). Down-regulation of p300/CBP histone acetyltransferase activates a senescence checkpoint in human melanocytes. Cancer Res.

[B41] Bandyopadhyay D, Curry JL, Lin Q, Richards HW, Chen D, Hornsby PJ, Timchenko NA, Medrano EE (2007). Dynamic assembly of chromatin complexes during cellular senescence: implications for the growth arrest of human melanocytic nevi. Aging Cell.

[B42] Gupta GP, Massagué J (2006). Cancer metastasis: building a framework. Cell.

