# Supplementary figures and images for: 14-3-3σ gene silencing during melanoma progression and its role in cell cycle control and cellular senescence
Source: Mol Cancer. 2009 Jul 30;8:53. doi: 10.1186/1476-4598-8-53 (PMC2723074; doi:10.1186/1476-4598-8-53)

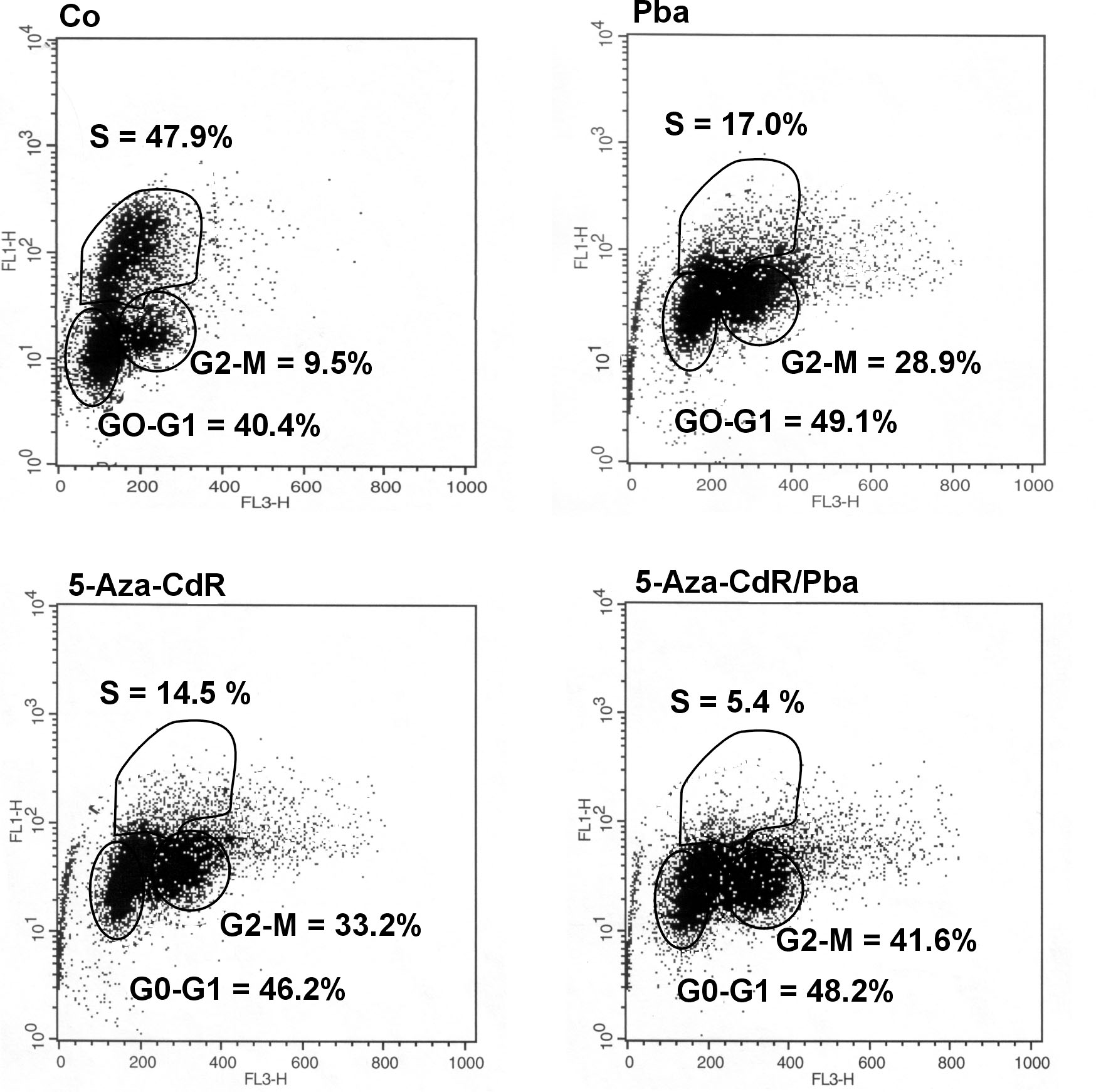

Supplement: Additional File 1 — Treatment of melanoma cells with 5-Aza-CdR, Pba, and 5-Aza-CdR/Pba, respectively, leads to inhibition of cell cycle progression. Cell cycle analysis of SK-Mel-147 melanoma cells was performed by measurement of BrdU uptake using flow cytometry after treatment of cells with 5-Aza-CdR, Pba, and 5-Aza-CdR/Pba, respectively. Flow cytometric profiles (dot blots) of one representative experiment are shown. [file 1476-4598-8-53-S1.jpeg]

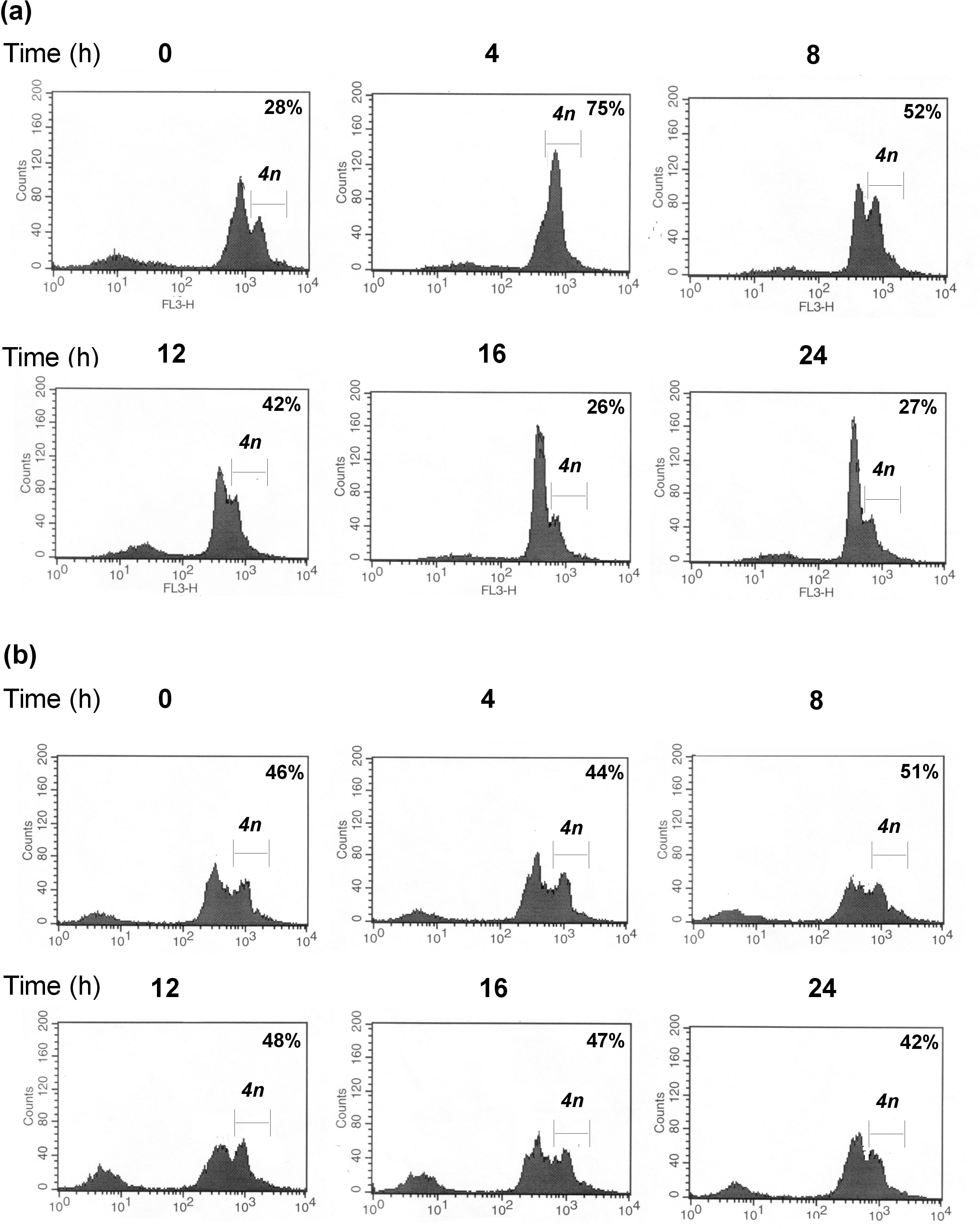

Supplement: Additional File 2 — Cell cycle progression of SK-Mel-147 cells after release of double thymidine bloc. (a) Synchronized, control (empty vector) transduced SK-Mel-147 cells were released from double thymidine block and the number of 4n DNA containing cells was analyzed by flow cytometry. (b) Synchronized, 14-3-3σ overexpressing SK-Mel-147 cells were released from double thymidine block and were analyzed as in (a). Histogram data from one representative experiment are shown in (a) and (b). Numbers in the diagrams indicate the percentages of 4n DNA containing cells. [file 1476-4598-8-53-S2.jpeg]
