# Supplementary material for: 14-3-3σ gene silencing during melanoma progression and its role in cell cycle control and cellular senescence
Source: Mol Cancer. 2009 Jul 30;8:53. doi: 10.1186/1476-4598-8-53 (PMC2723074; doi:10.1186/1476-4598-8-53)
Supplement: Additional File 3 — Histopathological data and clinical stage of primary melanomas analyzed by quantitative real-time PCR for 14-3-3σ expression. 1) NMM, nodular malignant melanoma; SSM, superficial spreading malignant melanoma; LMM, lentigo maligna melanoma; ALM, acro-lentiginous malignant melanoma. 2) Classification of clinical stage was done according to Balch CM, Buzaid AC, Atkins MB, Cascinelli N, Coit DG, Fleming ID, Houghton Jr. A, Kirkwood JM, Mihm MF, Morton DL, Reintgen D, Ross MI, Sober A, Soong SJ, Thompson JA, Thompson JF, Gershenwald JE, McMasters KM. A new American Joint Committee on Cancer staging system for cutaneous melanoma. Cancer 2000;88: 1484–91. [file 1476-4598-8-53-S3.doc]

**Supplemental Table 1** Histopathological data and clinical stage of primary melanomas analyzed by quantitative real-time PCR for 14-3-3σ expression

| **Number** | **Subtype ^1)^** | **Vertical tumor thickness** | **Clark Level** | **Clinical stage ^2)^** |
| --- | --- | --- | --- | --- |
| 1. | NMM | 4.2 mm | L IV | IIB |
| 2. | NMM | 11.0 mm | L V | IIC |
| 3. | NMM | 2.5 mm | L IV | IIA |
| 4. | NMM | 5.4 mm | L IV | IIC |
| 5. | SSM | 3.1 mm | L IV | IIB |
| 6. | SSM | 2.4 mm | L III | IIA |
| 7. | SSM | 4.8 mm | L IV | IIB |
| 8. | NMM | 3.1 mm | L III | IIB |
| 9. | SSM | 0.65 mm | L III | IA |
| 10. | SSM | 1.4 mm | L III | IB |
| 11. | LMM | 4.16 mm | L III | IIB |

^1)^ NMM, nodular malignant melanoma; SSM, superficial spreading malignant melanoma; LMM, lentigo maligna melanoma; ALM, acro-lentiginous malignant melanoma

^2^) Classification of clinical stage was done according to Balch CM, Buzaid AC, Atkins MB, Cascinelli N, Coit DG, Fleming ID, Houghton Jr. A, Kirkwood JM, Mihm MF, Morton DL, Reintgen D, Ross MI, Sober A, Soong SJ, Thompson JA, Thompson JF, Gershenwald JE, McMasters KM. A new American Joint Committee on Cancer staging system for cutaneous melanoma. Cancer 2000;88: 1484-91
