# Supplementary material for: 14-3-3σ gene silencing during melanoma progression and its role in cell cycle control and cellular senescence
Source: Mol Cancer. 2009 Jul 30;8:53. doi: 10.1186/1476-4598-8-53 (PMC2723074; doi:10.1186/1476-4598-8-53)
Supplement: Additional File 4 — Histopathological data and clinical stage of primary melanomas analyzed by methylation-specific PCR. Table showing histopathological data and clinical stage of primary melanomas analyzed by methylation-specific PCR. 1)/2) Melanoma subtypes and classification of clinical stage are explained in text of Supplemental Table 1. [file 1476-4598-8-53-S4.doc]

**Supplemental Table 2** Histopathological data and clinical stage of primary melanomas analyzed by methylation-specific PCR

| **Number** | **Subtype ^1)^** | **Vertical tumor thickness** | **Clark Level** | **Clinical stage ^2)^** |
| --- | --- | --- | --- | --- |
| 1. | SSM | 0,64 mm | L II | IA |
| 2. | SSM | 2,48 mm | L III | IIA |
| 3. | SSM | 1.76 mm | L III | IB |
| 4. | SSM | 4,41 mm | L IV | IIB |
| 5. | SSM | 1,2 mm | L III | IB |
| 6. | NMM | 2.95 mm | L III | IIA |
| 7. | NMM | 2.7 mm | L III | IIA |
| 8. | SSM | 1.22 mm | L III | IB |
| 9. | SSM | 1.1 mm | L II | IIA |
| 10. | SSM | 0.65 mm | L III | IA |
| 11. | NMM | 3.1 mm | L IV | IIC |
| 12. | NNM | 1.7 mm | L IV | IIA |
| 13. | SSM | 2.4 mm | L III | IIB |
| 14. | NNM | 3.1 mm | L III | IIC |
| 15. | SSM | 0.85 mm | L III | IA |

^1)^ / ^2)^ Melanoma subtypes and classification of clinical stage are explained in text of Supplemental Table 1.
